# *Chlamydia trachomatis* induces the transcriptional activity of host YAP in a Hippo-independent fashion

**DOI:** 10.3389/fcimb.2023.1098420

**Published:** 2023-02-27

**Authors:** Liam T. Caven, Amanda J. Brinkworth, Rey A. Carabeo

**Affiliations:** ^1^ Department of Pathology and Microbiology, University of Nebraska Medical Center, Omaha, NE, United States; ^2^ School of Molecular Biosciences, College of Veterinary Medicine, Washington State University, Pullman, WA, United States

**Keywords:** *Chlamydia trachomatis*, host response, YAP, pathogen-directed host transcription, Hippo kinase

## Abstract

**Introduction:**

The obligate intracellular pathogen Chlamydia trachomatis is the causative agent of the most common bacterial sexually transmitted disease worldwide. While the host response to infection by this pathogen has been well characterized, it remains unclear to what extent host gene expression during infection is the product of Chlamydia-directed modulation of host transcription factors.

**Methods:**

To identify transcription factors potentially modulated by Chlamydia during infection, we infected immortalized endocervical epithelial cells (End1/E6E7) with the anogenital C. trachomatis serovar L2, harvesting polyadenylated RNA for bulk RNA-sequencing. Subsequent experiments elucidating the mechanism of infection-mediated YAP activation assayed YAP target gene expression via qRT-PCR, YAP nuclear translocation via quantitative immunofluorescence, and YAP phosphorylation via Western blotting.

**Results:**

RNA sequencing of Chlamydia-infected endocervical epithelial cells revealed gene expression consistent with activity of YAP, a transcriptional coactivator implicated in cell proliferation, wound healing, and fibrosis. After confirming induction of YAP target genes during infection, we observed an infection-dependent increase in YAP nuclear translocation sensitive to inhibition of bacterial protein synthesis. While Hippo-mediated phosphoinhibition of YAP at S127 was unaffected by C. trachomatis infection, Hippo-independent phosphorylation at Y357 was increased. Infection did not enhance nuclear translocation of Y357F mutant YAP, illustrating a requirement for phosphorylation at this residue. Pharmacological inhibition of host Src-family kinase activity attenuated YAP Y357 phosphorylation, but not nuclear translocation – which was instead sensitive to inhibition of Abl.

**Discussion:**

Our results define a transcriptome-altering mechanism of pathogen-directed YAP activation that bypasses canonical inhibition by the Hippo kinase cascade, with a potential link to chlamydial fibrosis and other advanced disease sequelae. Additional study is required to determine the specific role of infection-associated Y357 phosphorylation and Abl activity in chlamydial induction of YAP.

## 1 Introduction

*Chlamydia trachomatis* is the most common bacterial cause of both sexually-transmitted disease and infectious blindness worldwide. An obligate intracellular pathogen, *C. trachomatis* binds to and induces its own internalization by epithelial cells, establishing a replicative niche within a pathogen-remodeled vacuole termed the chlamydial inclusion. Chronic or repeated *C. trachomatis* infection is associated with severe inflammatory and fibrotic sequelae. *C. trachomatis* serovars A-C, which preferentially infect the conjunctiva, are associated with excess collagen production and contraction of infected tissues, leading to the inward turning of the upper eyelid and subsequent corneal abrasion by the eyelashes (Taylor et al., 2014; Lansingh, 2016). *Chlamydia*-mediated trachoma is endemic to Africa, Latin America, Asia, and the Middle East (Hu et al., 2010), and is responsible for the blindness or visual impairment of up to 1.9 million people according to current WHO estimates. Infection by *C. trachomatis* serovars D-K, which preferentially colonize the reproductive tract, are associated with pelvic inflammatory disease as well as progressive scarring of the fallopian tubes (Ness et al., 2008; Haggerty et al., 2010). Blockage of the upper female genital tract by scar tissue can consequently lead to ectopic pregnancy or tubal factor infertility (Chow et al., 1990). In the United Kingdom, *C. trachomatis* is estimated to account for 29% of all cases of tubal factor infertility, which in turn represents 25% of all cases of female infertility (Price et al., 2016). Collectively, the urgent threat to public health presented *Chlamydia*-associated sequelae has made understanding the underlying mechanisms of the host-*Chlamydia* interaction a subject of ongoing investigation.

Initial study of the host response to infection has made a persuasive argument for infection-associated pathology being the product of cytokine signaling cascade originating in infected epithelial cells, termed the cellular paradigm of pathogenesis (Stephens, 2003). Clinical infections have revealed substantial infiltration of infected tissues by immune cells *in vivo*, suggesting that infected epithelial cells recruit components of the innate and adaptive immune systems to the site of infection (Kiviat et al., 1990; Stephens, 2003). Indeed, upregulation of an expansive portfolio of inflammatory cytokines has been consistently observed in chlamydial infections *in vitro*, including GROα, GM-CSF, IL-1α, IL-6, IL-11, and IL-8 (Eckmann et al., 1993; Rasmussen et al., 1997; Cheng et al., 2008). However, the pro-inflammatory host response fails to account for the fibrotic outcomes of asymptomatic infection; it is estimated that up to 75% of *C. trachomatis* infections go unreported due to absent or subclinical symptoms, and that up to 18% of such cases are believed to cause infertility (Haggerty et al., 2010).

One explanation for this discrepancy in the data is that *C. trachomatis* induces pathology *via* direct manipulation of host gene expression; indeed, mounting evidence suggests that regulation of host transcription is targeted by the pathogen in a variety of ways. Past work has shown that *C. trachomatis* antagonizes pro-inflammatory signaling mediated by the transcription factor NF-κB by stabilizing its inhibitory subunit IκBα, through the activity of the chlamydial deubiquitinase ChlaDub1 (Le Negrate et al., 2008). A report that ectopic expression of the chlamydial protease CPAF leads to degradation of the NF-κB complex subunit p65/R suggested a similar role for this effector in modulation of host gene expression. However, the relevance of CPAF to NF-κB antagonism has been brought into question by new data, given that harvest of host protein lysates with more robust methods of CPAF inhibition did not result in p65 cleavage (Johnson et al., 2015), and infection with CPAF-deficient mutant strains does not exhibit increased NF-κB activation (Snavely et al., 2014). That said, a more recent report proposed chlamydial effectors delivered by the pathogen’s type III secretion system may yet cooperate with CPAF to inhibit p65 nuclear translocation (Patton et al., 2016). Combined with the activity of ChlaDub1 on IκKα, these data imply *C. trachomatis* likely antagonizes NF-κB *via* multiple, complementary mechanisms. Infection with the related species *C. pneumonia*e has been shown to induce expression and phosphorylation of the AP-1 transcription factor c-Jun (Krämer et al., 2015). Intriguingly, this effect did not occur during infection with heat-killed *C. pneumoniae*, indicating that c-Jun activation is a pathogen-directed phenotype with implications for virulence, given that c-Jun knockdown negatively impacted bacterial load as well. Collectively, these data illustrate the potential for *Chlamydiae* to induce changes in host gene expression to facilitate pathogenesis, demonstrating a need to better characterize how the pathogen acts on host transcription factors.

To that end, we have examined the host transcriptome of *C. trachomatis*-infected immortalized endocervical epithelial cells, to perform unbiased *in silico* discovery of pathogen*-*modulated host transcription factors. In this report, we show that *C. trachomatis* infection induces gene expression consistent with the function of YAP (Yes-associated protein), a transcriptional coactivator. We observed infection-dependent increases in YAP nuclear incidence and phosphorylation at Y357; this post-translational modification was shown previously to enhance YAP activation and increased transcription of target genes (Smoot et al., 2018; Sugihara et al., 2018). YAP Y357 phosphorylation in infected cells was attenuated by treatment with inhibitors of Src-family kinases (SFKs); by contrast, nuclear incidence of YAP was sensitive to inhibition of Abl. With these data, we present an alternative mechanism of enhancing YAP activation by an intracellular pathogen, independent of targeting the Hippo kinase cascade, and potentially underpinning a pathogenic strategy by *Chlamydia trachomatis* to drive changes in host gene expression by manipulating transcription factor activity.

## 2 Materials and methods

### 2.1 Eukaryotic cell culture

Human endocervical epithelial HPV-16 E6/E7 transformed End1s (End1 E6/E7, ATCC CRL-2615) were cultured at 37° C with 5% atmospheric CO_2_ in Keratinocyte Serum-Free Medium (KSFM, Thermo Fisher Scientific) supplemented with human recombinant epidermal growth factor, bovine pituitary extract, 5 micrograms/mL gentamicin, and 0.4 mM CaCl_2_ (unless otherwise indicated). For all experiments, End1s were cultured between passages 3 and 15. Primary human cervical epithelial cells (HCECs, ATCC PCS-0480-011, Lot 80306190) were cultured at 37° C with 5% atmospheric CO_2_ in Cervical Epithelial Cell Basal Medium (CECBM, ATCC PCS-480-032) supplemented with all contents of a Cervical Epithelial Growth Kit (ATCC PCS-080-042). Raft cultures were prepared as previously described by our laboratory (Nogueira et al., 2017), exchanging HaCaT cells for End1s where indicated, and cultured over 19 days before being fixed for 30 minutes in 4% paraformaldehyde, OCT-embedded, cryo-sectioned, and stained with hematoxylin and eosin.

### 2.2 Cloning and DNA/siRNA transfection

Both FLAG-YAP1-WT and FLAG-YAP1-Y357F were generated *via* the simplified FastCloning method of Gibson assembly (Li et al., 2011); briefly, PCR-amplified recombinant DNA was treated with DpnI (NEB Biolabs) for 3 h, then transformed into Stellar chemically competent *E. coli* (Clontech 636763) *via* heat shock, followed by Kanamycin selection, screening *via* colony PCR (where applicable), and sequence confirmation. FLAG-YAP1-WT was generated *via* amplification of pEGFP-C3-HYAP1, a gift from Marius Sudol (Addgene plasmid # 17843; http://n2t.net/addgene:17843; RRID : Addgene_17843), using the following primers: FWD: 5’-GACTACAAAGACGATGACGACAAGGATCCCGGGCAGCAGCCG-3’; REV: 5’-CTTGTCGTCATCGTCTTTGTAGTCCATGGTGGCGACCGGTAGCG-3’. FLAG-YAP1-Y357F was generated from FLAG-YAP1-WT *via* site-directed mutagenesis of the Y357 codon (TAC to TTC), using the following primers: FWD: 5’-GCAGCTTCAGTGTCCCTCGA-3’, REV: 5’- ACACTGAAGCTGCTCATGCTTAGTC-3’.

End1s were transfected with plasmid DNA *via* electroporation: after harvest *via* trypsinization, 2x10^6^ End1s per construct were resuspended in 400 μL Opti-MEM at room temperature (Thermo Fisher Scientific 31985062) containing 20 ug of plasmid DNA and 3 μL sheared salmon sperm DNA (Thermo Fisher Scientific AM9680). Suspensions were then transferred to individual GenePulser 0.4 cm cuvettes (Bio-Rad 1652088), then electroporated using a GenePulser XCell (Bio-Rad) using the following parameters: exponential decay program template, 225 V, 850 μF capacitance, infinite resistance. 100 μL aliquots of the resulting electroporants were transferred to individual wells of a 24-well plate containing cover slips and pre-warmed KSFM, then incubated until confluent monolayers had formed (24-48h) for subsequent infection as previously described.

For siRNA-mediated knockdown experiments, End1s were transfected with either an ON-TARGETplus non-targeting siRNA pool (Horizon Discovery D-001810-10-05) or an ON-TARGETplus YAP1-targeting siRNA SMARTpool (Horizon Discovery L-012200-00-0005) using Lipofectamine 3000 (Thermo Fisher Scientific L3000008), per manufacturer’s instructions, at an empirically determined optimal concentration of 10 nM. At 16 h post-seeding of End1s at 125% of confluence on 6-well plates as described above, siRNA was combined in Opti-MEM (Thermo Fisher Scientific 31985062) with the Lipofectamine 3000 reagent, incubated for 5 m at room temperature to allow for liposome formation, then added to wells dropwise with mixing. Transfected End1s were then incubated for 24 h prior to infection with *Chlamydia* (see below).

### 2.3 Chlamydial infections

*Chlamydia trachomatis* serovar L2 (434/Bu) was originally obtained from Dr. Ted Hackstadt (Rocky Mountain National Laboratory, NIAID). Chlamydial EBs were isolated from infected, cycloheximide-treated McCoy cells at 36-40 hours post-infection (hpi) and purified by density gradient centrifugation as previously described (Caldwell et al., 1981). For infection on 6- and 24-well tissue culture plates (Greiner Bio-One 657160 and 662160), End1s were seeded at 125% of confluence to ensure uniform formation of monolayers occurred in the event of any cell death prior to infection. HCECs were grown to confluence after seeding at a density of 5000 cells/cm^2^, per manufacturer’s recommendations. End1 (16 h post-seeding) and HCEC monolayers (6-7 days post-seeding) were washed with pre-warmed Hanks Buffered Saline Solution (HBSS) prior to inoculation with *Chlamydia*-containing KSFM or CECBM. To ensure uniformity of infection between individual cells for measurement of YAP nuclear translocation and/or phosphorylation, all infections were carried out at a multiplicity of infection (MOI) of 5, unless otherwise indicated. Tissue culture plates were centrifuged at 4° C and 500 rcf (Eppendorf 5810 R tabletop centrifuge, A-4-81 rotor) for 15 minutes to synchronize infection. Inoculum was then aspirated, and cells were washed with chilled HBSS prior to the addition of pre-warmed KSFM or CECBM.

Where indicated, media was replaced with fresh, pre-warmed KSFM containing DMSO (Thermo Fisher Scientific D12345, 1:1000), verteporfin (Cayman Chemical Company 17334, 5 μM), chloramphenicol (Sigma-Aldrich C0378, 50 μg/mL), sodium orthovanadate (Sigma-Aldrich 450243, 5 μM), RK-24466 (Cayman Chemical Company 15135, 10 nM), or PP2 (EMD Millipore 529576, 10 μM). Infected cultures were then returned to the tissue culture incubator until the indicated times post-infection.

### 2.4 Bulk RNA-sequencing and analysis

Prior reports of host gene expression during *C. trachomatis* infection have employed an MOI of 1 (Humphrys et al., 2013; Porcella et al., 2015); given the potential for low MOI to produce a heterogeneous population of infected and uninfected cells, we opted to employ an MOI of 2 as a compromise between consistency with past work and ensuring uniformity of infection (and, thereby, identification of host transcription factors modulated by infection). Thus, End1s seeded on fibronectin-coated 6-well plates (Corning 354402) were infected at an MOI of 2 as described above (pre/post-washing, 15 m centrifugation at 500 rcf and 4° C) and harvested for RNA using TRIzol (Thermo Fisher Scientific 15596026) and the DNA-free DNA removal kit (Thermo Fisher Scientific AM1906), according to manufacturers’ protocols. Extracted RNA was subsequently enriched for polyadenylated mRNA transcripts using the NucleoTrap mRNA enrichment kit (Macherey-Nagel) according to the manufacturer’s protocol. For the comparison of mock- and *Ct* serovar L2-infected transcriptomes, RNA samples were subsequently assayed for fragmentation using an Agilent 5200 Fragment Analyzer; intact samples were enriched for polyadenylated transcripts *via* the NucleoTrap kit a second time due to the high incidence of ribosomal RNA in the fragment analysis results. cDNA library preparation was performed using the Ion Xpress Plus Fragment Library kit (Thermo Fisher Scientific 4471269), and sequencing of cDNA libraries was performed using the Ion Torrent system (Life Technologies). For the bulk RNA-sequencing of primary cells, HCECs infected at an MOI of 2 as described above (pre/post-infection washing, 15 m centrifugation at 500 rcf and 4° C) were harvested for RNA samples *via* TRIzol and the DNA-free DNA removal kit as described above, and were subsequently assayed for fragmentation using an Agilent Bioanalyzer 2100. cDNA library preparation of intact samples was performed using the NuGEN Universal mRNA-Seq Library Preparation kit, and sequencing of cDNA libraries was performed using the NextSeq 550 system (Illumina).

Read alignment and downstream analysis of both experiments was performed using CLC Genomics Workbench (Qiagen); each treatment group was comprised of libraries from three biological replicates, each with a minimum of 30 million reads (unstranded single read, mean length 150 bp), genes with an FDRP ≤ 0.05 were considered differentially expressed. ChEA crossreferencing was performed in Python, identifying the number of genes induced (FC ≥ 1.5, FDRP ≤ 0.05) and repressed (FC ≤ 1.5, FDRP ≤ 0.05) by infection for each transcription factor’s gene targets (Lachmann et al., 2010). Pearson’s correlation coefficients between End1 and HCEC expression of YAP-responsive genes were subsequently calculated in R, using log_2_-transformed fold changes of all genes differentially expressed in at least one data set. GO-BP term enrichment analysis was performed in R using the clusterProfiler gene set enrichment analysis package (Yu et al., 2012). The bulk RNA-sequencing datasets generated and analyzed for this study can be found in the Gene Expression Omnibus (Edgar et al., 2002), GEO Series accession number GSE180784 (https://www.ncbi.nlm.nih.gov/geo/query/acc.cgi?acc=GSE180784).

### 2.5 Reverse transcription quantitative real-time PCR

End1s seeded on fibronectin-coated 6-well plates (Corning 354402) and infected at an MOI of 5 as described above (pre/post-infection washing, 15 m centrifugation at 500 rcf and 4° C) were harvested for RNA using TRIzol (Thermo Fisher Scientific 15596026) and the DNA-free DNA removal kit (Thermo Fisher Scientific AM1906), according to manufacturers’ protocols. cDNA libraries were subsequently prepared using SuperScript IV Reverse Transcriptase (Thermo Fisher Scientific 11766050) according to the manufacturer’s protocol. Quantitative real-time PCR was performed on a QuantStudio 3 (Thermo Fisher Scientific) using TaqMan assay kits (Thermo Fisher Scientific) of the following genes: CTGF (Hs01026927_g1), CYR61 (Hs00998500_g1), INHBA (Hs01081598_m1), BMP2 (Hs00154192_m1), and the housekeeping gene HPRT (Hs02800695_m1). Statistical analysis was performed in R, using pairwise Student’s t-tests and Bonferroni’s correction for multiple comparisons; p-values less than 0.05 were considered statistically significant.

### 2.6 Immunofluorescence microscopy

End1s or HCECs seeded on glass cover slips (VWR) coated with fibronectin (Corning 354008) or fibronectin-deposited micropatterned chips (CYTOO) and infected at an MOI of 5 as described above (pre/post-infection washing, 15 m centrifugation at 500 rcf and 4° C) were fixed in 4% paraformaldehyde in phosphate-buffered saline (PBS) for 10 minutes at 37° C. After permeabilization *via* a 5 m incubation in PBS containing 0.25% Triton X-100, cover slips were washed in PBS, then blocked in 5% bovine serum albumin (BSA) in PBS for 1 hour at room temperature. Fixed and blocked cover slips were subsequently incubated overnight at 4° C with primary antibodies in 1% BSA-PBS: mouse anti-*Ct* Hsp60 (Invitrogen MA3-023, 1:250 dilution), goat anti-MOMP (Meridian Life Science B65266G, 1:1000 dilution), rabbit anti-YAP (CST 4912, 1:100 dilution), rabbit anti-E-Cadherin (CST 3195, 1:200 dilution). Cover slips were again washed in PBS, then incubated for 1 hour at room temperature with the following fluorophore-conjugated antibodies/dyes in 1% BSA-PBS: donkey anti-mouse Alexa-488 conjugate (Thermo Fisher Scientific A-21202, 1:1000 dilution), chicken anti-goat Alexa-594 conjugate (Thermo Fisher Scientific A-21468, 1:1000 dilution), goat anti-rabbit Alexa-488 conjugate (Thermo Fisher Scientific A-11034, 1:1000 dilution), phalloidin Alexa-594 conjugate (Thermo Fisher Scientific A-12381, 1:120 dilution), DAPI (Sigma-Aldrich 10236276001, 1:1000 dilution). Afterward, cover slips were washed in PBS and ultrapure water, then mounted on microscope slides using Shandon Immu-Mount (ThermoFisher Scientific 9990402).

A minimum of 5 fields per cover slip were imaged using a SP-8 Lightning Confocal Microscope (Leica) or CSU-W1 Spinning-Disk Confocal Microscope (Nikon). To account for variations in nuclear size and avoid biased selection of cells for measurement, blinded image quantification was performed by assigning image filenames randomized number codes, selecting 10 nuclei at random per field using only the DAPI channel, manually masking the nuclear area, and recording the mean YAP fluorescence intensity per nucleus. To account for variation in total YAP between cells, staining efficiency between cover slips, and compression of the nuclear/cytosolic compartments by the chlamydial inclusion, 5 cytosolic regions not occluded by an inclusion body and adjacent to measured nuclei were selected per field, with the mean YAP fluorescence intensity of these regions averaged to produce a per-field measurement of mean cytosolic YAP fluorescence intensity; nuclear translocation of YAP was thereby expressed as a ratio of mean nuclear fluorescence to mean cytosolic fluorescence. Statistical analysis was performed in R, using a Kruskal-Wallis test to first verify a statistically significant (p-value < 0.05) difference between treatment groups. Subsequent pairwise comparisons were performed using a Wilcoxon rank sum test and Bonferroni’s correction for multiple comparisons, with p-values less than 0.05 being considered statistically significant.

### 2.7 SDS-PAGE and western blotting

To minimize activity of the chlamydial protease CPAF, End1s seeded on 6-well plates and infected at an MOI of 5 as described above (pre/post-infection washing, 15 m centrifugation at 500 rcf and 4° C) were subsequently lysed in 1% SDS buffer heated to 95° C, as previously described (Johnson et al., 2015). After treatment with Pierce Universal Nuclease (Thermo Fisher Scientific, 1:1000 dilution) for 5 minutes at room temperature, lysates were combined with 4X Laemmli Sample Buffer (Bio-Rad 1610747) for loading on a 7.5% acrylamide/bis-acrylamide gel for SDS-PAGE (1.5 hours, 120V). Gels were then transferred to PVDF membranes (Bio-Rad 1620177) using a semi-dry transfer method. After blocking in 5% BSA in PBST (PBS containing 0.1% Tween-20) for 1 hour at room temperature, membranes were incubated overnight in 5% BSA-PBST at 4° C with primary antibodies: rabbit anti-YAP (CST 4912, 1:1000 dilution), rabbit anti-S127-pYAP (CST 13008, 1:1000 dilution), rabbit anti-Y357-pYAP (Abcam ab62571, 1:1000 dilution), mouse anti-*Ct* Hsp60 (Invitrogen MA3-023, 1:1000 dilution in). Membranes were subsequently washed in PBST, then incubated in 5%-BSA PBST with either a goat anti-rabbit HRP-conjugated secondary antibody (Dako P0448, 1:2000 dilution) or rabbit anti-mouse HRP-conjugated secondary antibody (Dak P0161, 1:4000 dilution) for 2 hours at room temperature. After additional washing in PBST, membranes were imaged using Immobilon HRP Substrate (Millipore Sigma WBKLS0500) or an Azure Biosystems c600. Images were analyzed using the ImageJ gel analysis tool to quantify the fluorescence density of phospho-analyte bands relative to the YAP total protein loading control. Statistical analysis was performed in R, using pairwise Student’s t-tests with Bonferroni’s correction for multiple comparisons; p-values less than 0.05 were considered statistically significant.

## 3 Results

### 3.1 *Chlamydia* infection induces the expression of a subset of YAP target genes

**Figure 1:**
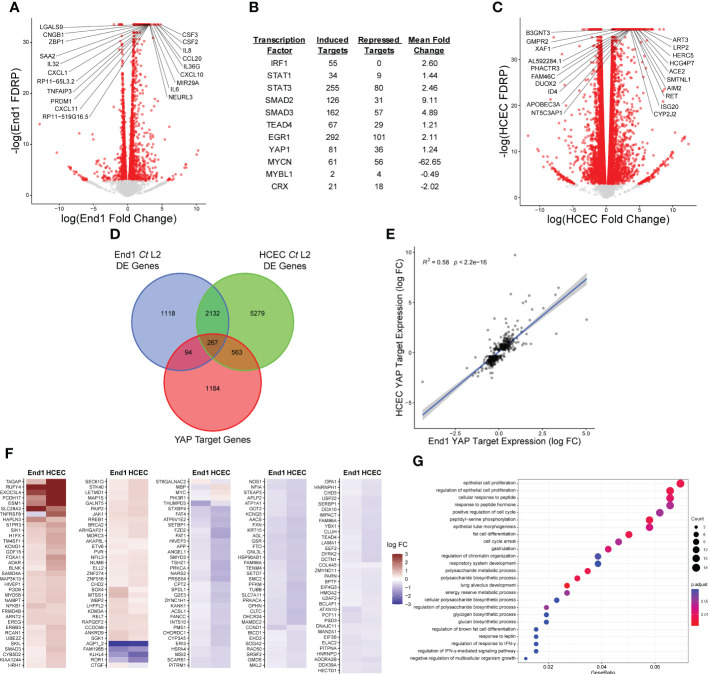
*Chlamydia* infection induces expression of a subset of YAP target genes. **(A)** Volcano plot of gene expression in bulk RNA-sequencing of End1/E6E7 immortalized epithelial cells (End1s) during infection with *Chlamydia trachomatis* (*Ct*) serovar L2 compared to mock infection, at 24 hours post-infection (hpi). n = 3, with a minimum of 3x10^7^ unstranded single reads per replicate with a mean length of 150 bp. All fold changes are relative to the mock-infected control; red dots: false discovery rate p-value (FDRP) ≤ 0.05, labels: top 20 genes whose expression differed most significantly (lowest FDRP) from the mock infection. **(B)** Table of selected transcription factors identified as potential targets of infection-associated modulation *via* cross-referencing of differentially expressed genes identified in **(A)** with the ChIP Enrichment Analysis (ChEA) database of transcription factor target genes. See also Supplementary Data S2. **(C)** Volcano plot of gene expression in bulk RNA-sequencing of primary human endocervical epithelial cells (HCECs) during infection with *Ct* serovar L2 compared to mock infection, at 24 hours post-infection (hpi). n = 3, with a minimum of 3x10^7^ unstranded single reads per replicate with a mean length of 150 bp. All fold changes are relative to the mock-infected control; red dots: false discovery rate p-value (FDRP) ≤ 0.05, labels: top 20 genes whose expression differed most significantly (lowest FDRP) from the mock infection. **(D)** Venn diagram of differentially expressed genes identified in **(A, C)** cross-referenced with the ChEA database of YAP target genes. **(E)** Scatter plot of gene expression of YAP-responsive (ChEA), differentially expressed genes in either *Ct* serovar L2-infected End1s (x-axis) or HCECs (y-axis). All fold changes are relative to each cell type’s respective mock-infected control; blue line: linear regression model of correlation; grey shading: 95% confidence interval. R^2^ and p-values calculated using Pearson’s correlation. **(F)** Heatmap of YAP target gene expression in *Ct* serovar L2-infected End1s (left columns) and HCECs (right columns). All fold changes are relative to each cell type’s respective mock-infected control; only genes differentially expressed (FDRP ≤ 0.05) in both cell types are shown. **(G)** Dot plot of GO biological process term enrichment in the set of YAP target genes differentially expressed in *Ct* serovar L2 infection of End1s or HCECs (top 25 most significantly enriched terms shown). Dot size: number of term-associated genes found in set, dot color: adjusted p-value.

To identify host transcription factors modulated by *Chlamydia* infection, we opted to model infection of the upper female genital tract using End1/E6E7 immortalized, non-transformed endocervical epithelial cells (Fichorova et al., 1997). We first confirmed the non-tumorigenic growth properties of End1 cells *via* organotypic raft culture: first seeding End1 cells on collagen gels in a transwell insert, then inducing differentiation *via* exposure to the air-liquid interface (Nogueira et al., 2017). Transformed cervical epithelial cells form irregular layers when induced to differentiate *via* air-liquid interface (Aasen et al., 2003); by contrast, differentiating End1s grown *via* raft culture formed epithelial tissues of uniform thickness (**Supplementary Figure S1**), consistent with previous reports of organotypic culture with this cell line (Gali et al., 2010). Having thus validated the non-tumorigenic phenotype of our experimental model, we then infected End1s with the lymphogranuloma venereum *Chlamydia trachomatis* serovar L2, then harvested polyadenylated RNA for bulk RNA-sequencing at 24 hours post-infection (hpi). This timepoint occurs well after chlamydial differentiation into proliferative and metabolically active reticulate bodies (Lee et al., 2018), thus allowing for production of any chlamydial factors modulating host gene expression. That being said, factors produced by *C. trachomatis* at later stages of infection may also modulate host gene expression; thus, we cannot at this time dismiss the possibility of additional infection-mediated alterations to the host transcriptome occurring later than 24 hpi. Nevertheless, infection at this time point induced the differential expression (FDRP ≤ 0.05) of 3611 genes relative to a mock-infected control (**Figure 1A**; **Supplementary Data S1**, GEO Accession: GSE180784).

To then determine potential transcription factors driving the host response to infection, we cross-referenced this differentially expressed gene set with the ChEA database of transcription factor gene targets, derived from gene set enrichment analysis of published ChIP-chip, ChIP-seq, ChIP-PET, and DamID data (Lachmann et al., 2010). Of the 199 transcription factors included in ChEA, 149 exhibited twice as many induced target genes (FC ≥ 1.5) as repressed target genes (FC ≤ 1.5), suggesting a general chlamydial induction of transcription (**Supplementary Data S2**). Critically, targets of transcription factors known to regulate inflammation (IRF1, STAT1, STAT3) and fibrosis (SMAD2/3, EGR1, TEAD4, YAP1) appeared to be highly induced in the host (**Figure 1B**), consistent with prior reports of infection-dependent induction of pro-inflammatory and pro-fibrotic gene expression (Carlson et al., 2005; Lad et al., 2005; Humphrys et al., 2013; Sun et al., 2017).

Intriguingly, the fibrosis-associated transcription factors identified by this analysis (SMAD2/3, EGR1, and TEAD4) are known binding partners of the transcriptional coactivator YAP1 (Zagurovskaya et al., 2009; Zanconato et al., 2015; Szeto et al., 2016). Importantly, 81 YAP target genes (as identified by ChEA) were induced in *Chlamydia*-infected End1s, suggesting infection may modulate YAP activity. To assess the physiological relevance of apparent modulation of YAP target genes, we sequenced polyadenylated RNA from primary human endocervical epithelial cells (HCECs) infected with *Ct* serovar L2 (**Supplementary Data S3**, GEO Accession: GSE180784). Infected HCECs exhibited differential expression (FDRP ≤ 0.05) of 8241 genes (**Figure 1C**), of which 830 (10.1%) mapped to the ChEA database of YAP-responsive genes (**Figure 1D**). Expression of YAP-responsive genes by infected End1s and HCECs exhibited significant correlation (**Figure 1E**), suggesting that infection of these cell types activates a conserved, putatively YAP-mediated transcriptional program. *Chlamydia*-infected End1 cells and HCECs exhibited both induction and repression of YAP target genes (**Figure 1F**; **Supplementary Data S4**), in accordance with recent work indicating YAP can situationally act as a transcriptional corepressor (Kim et al., 2015; Hoxha et al., 2020; Ma et al., 2022). Functional characterization of YAP-associated gene expression in both infections *via* GO-BP (gene ontology, biological process) term enrichment analysis identified terms associated with regulation of epithelial cell proliferation and organ morphogenesis (**Figure 1G**), consistent with prior reports implicating YAP in these processes (Zhao et al., 2008; Zhao et al., 2010). Taken together, these data imply that *Chlamydia* infection targets the YAP regulon to impact a subset of the transcriptome of host epithelial cells.

### 3.2 *Chlamydia* infection promotes YAP nuclear translocation

**Figure 2:**
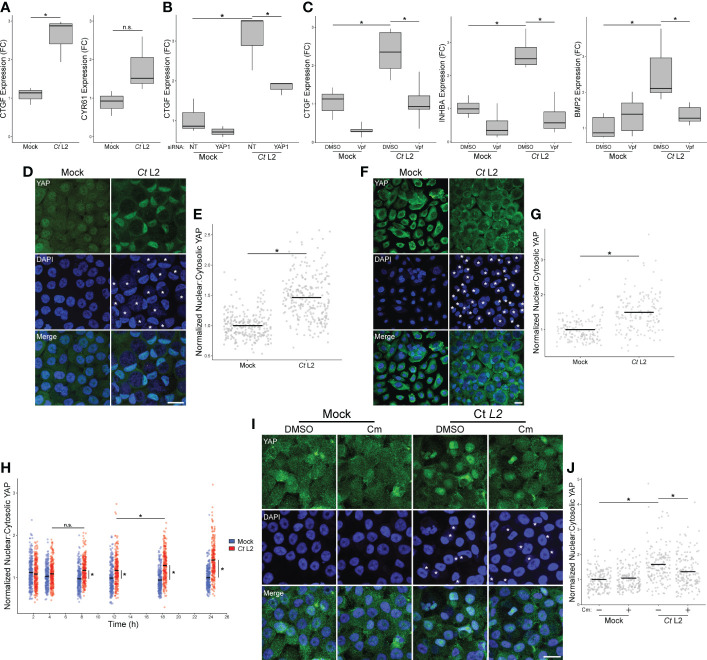
*Chlamydia* infection promotes YAP nuclear translocation. **(A)** Expression of CTGF and CYR61 at 24 hpi in mock- and *Ct* L2-infected End1 cells, as measured by RT-qPCR. n = 3 biological replicates; fold changes are relative to mean expression of the mock-infected and untreated control. Whiskers: minimum to maximum; n.s.: not significant (p-values > 0.05), asterisks: p-values ≤ 0.05, using pairwise Student’s t-tests and Bonferroni’s correction for multiple comparisons. **(B)** Expression of CTGF at 24 hpi in mock- and *Ct* L2-infected End1 cells transfected with non-targeting (NT) or YAP1-targeting siRNA (10 nM for 24 h prior to infection), as measured by RT-qPCR. n = 3 biological replicates; fold changes are relative to mean expression of the mock-infected and untreated control. Whiskers: minimum to maximum; asterisks: p-values ≤ 0.05, using pairwise Student’s t-tests and Bonferroni’s correction for multiple comparisons. **(C)** Expression of CTGF, INHBA, and BMP2 at 24 hpi in mock- and *Ct* L2-infected End1 cells treated with the YAP-TEAD inhibitor verteporfin (Vpf, 5 μM for 16 h starting at 8 hpi), as measured by RT-qPCR. n = 5 biological replicates; fold changes are relative to mean expression of the mock-infected and untreated control. Whiskers: minimum to maximum; asterisks: p-values ≤ 0.05, using pairwise Student’s t-tests and Bonferroni’s correction for multiple comparisons. **(D)** Representative micrographs of YAP (green) translocation into the nuclei (blue) of confluent mock- and *Ct* L2-infected End1 cells at 24 hpi. Asterisks: chlamydial inclusions, scale bar: 20 μm. **(E)** Quantification of YAP nuclear translocation in **(D)** as a ratio of nuclear to cytosolic YAP fluorescence. n = 5 biological replicates, 50 cells measured per sample. Black bars: group means; asterisks: p-values ≤ 0.05, using pairwise Wilcoxon rank sum tests and Bonferroni’s correction for multiple comparisons. **(F)** Representative micrographs of YAP nuclear translocation of confluent mock- and *Ct* L2-infected primary human cervical epithelial cells at 24 hpi. Asterisks, chlamydial inclusions, scale bar: 20 μm. **(G)** Quantification of YAP nuclear translocation in **(F)** as a ratio of nuclear to cytosolic YAP fluorescence. n = 5 biological replicates, 50 cells measured per sample. Black bars: group means; asterisks: p-values ≤ 0.05, using pairwise Wilcoxon rank sum tests and Bonferroni’s correction for multiple comparisons. **(H)** Quantification of YAP nuclear translocation at 2, 4, 8, 12, 18, and 24 hpi in confluent mock- and *Ct* L2-infected End1 cells as a ratio of nuclear to cytosolic YAP fluorescence. n = 5 biological replicates, 50 cells measured per sample. Blue dots: mock-infected cells, red dots: *Ct* L2-infected cells, black bars: group means, asterisks: p-value ≤ 0.05, using pairwise Wilcoxon rank-sum tests and Bonferroni’s correction for multiple comparisons. **(I)** Representative micrographs of YAP nuclear translocation at 18 hpi in confluent mock- and *Ct* L2-infected End1 cells treated with chloramphenicol (Cm, 50 μg/mL for 1 h at 17 hpi) or DMSO. Asterisks: chlamydial inclusions; scale bar: 20 μm. **(J)** Quantification of YAP nuclear translocation in **(I)**. n = 3 biological replicates, 50 cells measured per sample. Black bars: group means; asterisks: p-values ≤ 0.05, using pairwise Wilcoxon rank-sum tests and Bonferroni’s correction for multiple comparisons.

To confirm our RNA-sequencing analysis suggesting infection may promote YAP-dependent gene expression, we next assayed infection-dependent induction of known YAP target genes *via* RT-qPCR. Total RNA was harvested from confluent *C. trachomatis*-infected End1 cells at 24 hpi. Consistent with our bulk RNA sequencing results, infection increased expression of the direct YAP target CTGF (connective tissue growth factor), but not CYR61 (cysteine-rich angiogenic inducer 61) (**Figure 2A**), suggesting that infection potentially modulates the YAP regulon in a fashion distinct from previous reports of YAP activity. Critically, infection-dependent CTGF expression was sensitive to siRNA-mediated YAP knockdown (**Figure 2B**), confirming that chlamydial induction of this gene was YAP-dependent. A similar trend was exhibited by INHBA and BMP2 (**Supplementary Figure S2**) - genes induced by infection in our bulk RNA-seq results previously shown to be YAP-responsive (Mo et al., 2012; Huang et al., 2016). As incomplete YAP knockdown may be insufficient to inhibit expression of genes indirectly regulated by YAP, we then opted to treat infected cells with the YAP-TEAD complex inhibitor verteporfin (10 μM for 16 h, starting at 8 hpi). Consistent with our results with siRNA, verteporfin treatment significantly inhibited CTGF expression in *Chlamydia*-infected cells, as well as inhibiting expression of INHBA and BMP2 (**Figure 2C**). Taken together, these results suggest that a component of the infected host cell transcriptome may be driven by infection-associated alteration of the YAP regulon.

Transcriptional regulation by YAP of target genes requires nuclear translocation (Pocaterra et al., 2020). Thus, we opted to confirm that chlamydial infection promotes YAP activity by assaying the nuclear incidence of YAP in serovar L2-infected confluent End1 monolayers *via* immunofluorescence. Infection was performed at a multiplicity of 5 to ensure that the majority of cells were equivalently infected, and thus any infection-associated effects upon nuclear incidence of YAP would be uniform. To account for variations in nuclear size and staining efficiency, we performed blind quantification of randomly selected nuclei from infected cells in each sample, measuring ratios of nuclear to cytosolic YAP, analogous to previous study of YAP activation (Zhao et al., 2007; Dupont et al., 2011; Das et al., 2016). *C. trachomatis* infection of End1s was readily visualized by the presence of a vacuole containing DAPI-positive puncta adjacent to the host cell nucleus and colocalizing with staining of chlamydial Hsp60 and LPS (**Supplementary Figure S3**). Consistent with our initial observations of YAP target gene induction, nuclear YAP was increased in *Chlamydia*-infected End1/E6E7 cells at 24 hpi, relative to mock-infected control cells (**Figure 2D**). Indeed, infection was concomitant with a 1.5-fold increase in nuclear-to-cytosolic YAP ratio relative to mock-infected cells (**Figure 2E**). Given that expression of HPV protein E6 has been shown to stabilize YAP and thereby modulate its activity (Strickland et al., 2018), we assayed nuclear YAP in primary HPV-negative HCECs as well, observing an equivalent increase to that in E6-expressing End1s under similar infection conditions (**Figures 2F, G**). Collectively, these data indicate infection enhances YAP activation irrespective of HPV E6/E7 expression.

To determine if the observed increase in YAP activity was a *Chlamydia*-driven process requiring the action of one or more chlamydial effectors, we utilized chloramphenicol to inhibit chlamydial protein synthesis. Over the course of a 24-hour infection (at 2, 4, 8, 12, 18, and 24 hpi), we observed a significant increase in YAP nuclear localization by immunofluorescence between mock- and serovar L2-infected End1/E6E7 cells, starting as early as 8 hpi. A statistically significant increase between infected cells was first detected between 12 and 18 hpi, indicating that synthesis of YAP-modulating chlamydial effectors may occur at this stage of infection (**Figure 2H**). We next attempted to attenuate chlamydial YAP induction at 18 hpi *via* treatment with chloramphenicol (50 ug/mL). In agreement with our initial hypothesis, chloramphenicol treatment for one hour prior to fixation at 18 hpi was sufficient to significantly attenuate nuclear YAP relative to a DMSO-treated control (**Figures 2I, J**). Given these data, we conclude that YAP activation in infected cells requires *de novo* synthesis of chlamydial effectors.

### 3.3 *Chlamydia* infection bypasses Hippo-mediated YAP inhibition by enhancing YAP phosphorylation at Y357

**Figure 3:**
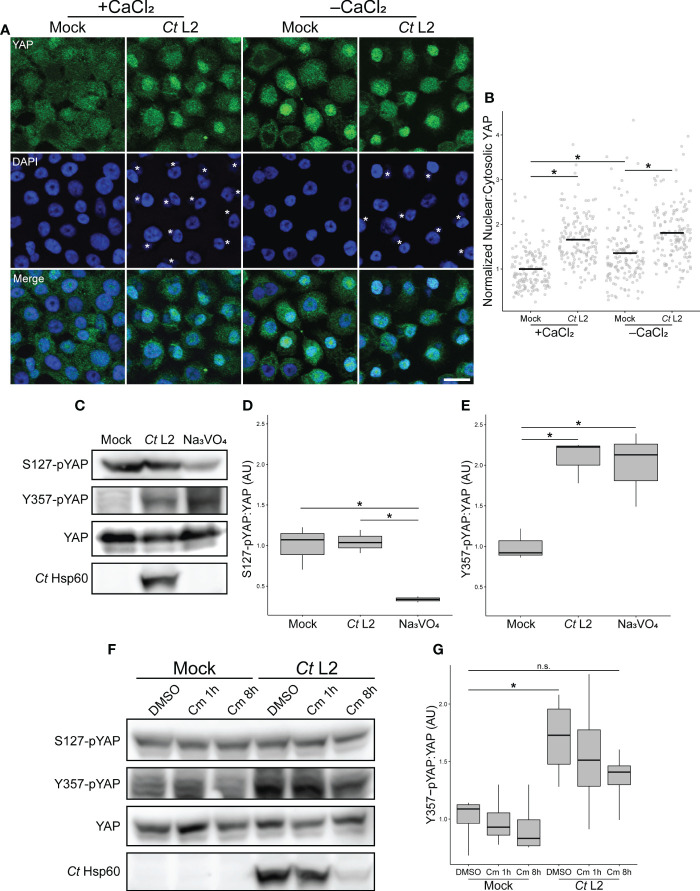
*Chlamydia* infection bypasses Hippo-mediated YAP inhibition by enhancing YAP phosphorylation at Y357. **(A)** Representative micrographs of YAP translocation (green) into the nuclei (blue) of confluent mock- and *Ct* L2-infected End1 cells at 24 hpi, cultured in calcium-replete (+CaCl_2_, 0.4 mM) or calcium-deplete (-CaCl_2_) keratinocyte serum-free media. Asterisks: chlamydial inclusions, scale bar: 20 μm. **(B)** Quantification of YAP nuclear translocation in **(A)** as a ratio of nuclear to cytosolic YAP fluorescence. n = 3 biological replicates, with a minimum of 5 fields measured per sample. Black bars: group means; asterisks: p-values ≤ 0.05, using pairwise Wilcoxon rank sum tests and Bonferroni’s correction for multiple comparisons. **(C)** Representative Western blot of YAP phosphorylation of mock- and *Ct* L2-infected or sodium orthovanadate-treated (Na_3_VO_4_, 10 mM for 1 h at 23 hpi) End1 cells at 24 hpi. **(D, E)** Densitometric quantification of YAP phosphorylation at S127 **(D)** and Y357 **(E)**, normalized to total YAP. n = 3 biological replicates; whiskers: minimum to maximum; asterisks: p-values ≤ 0.05, using pairwise Student’s t-tests and Bonferroni’s correction for multiple comparisons. **(F)** Representative Western blot of YAP phosphorylation of mock- and *Ct* L2-infected and DMSO or chloramphenicol-treated (Cm, 50 ug/mL for 1 h at 7 hpi or 8h at 10 hpi) End1 cells at 18 hpi. **(G)** Densitometric quantification of YAP Y357 phosphorylation, normalized to total YAP. n = 3 biological replicates; whiskers: minimum to maximum; n.s.: not significant (p-values > 0.05), asterisks: p-values ≤ 0.05, using pairwise Student’s t-tests and Bonferroni’s correction for multiple comparisons.

The Hippo kinase cascade is the major regulator of YAP activity in confluent epithelial cells (Gumbiner and Kim, 2014). When stabilized by cell-cell contact, Hippo initiates a Ser/Thr kinase cascade that leads to the phosphorylation of S127 residue of YAP, promoting cytosolic sequestration *via* binding to 14-3-3 (Piccolo et al., 2014). Increased nuclear YAP in infected cells could thus be due to chlamydial inhibition of the Hippo kinase cascade. To determine if chlamydial YAP activation was responsive to adherens junction disruption, we assayed nuclear incidence of YAP in two complementary conditions of attenuated cell-cell contact: small cell clusters seeded 70-85 μm apart on 30 μm (1-3 cells/cluster) and 45 μm (2-4 cells/cluster) fibronectin micropatterns (Degot et al., 2010), as well as confluent monolayers in which cell-cell contacts were disassembled *via* calcium withdrawal (O’Keefe et al., 1987). If YAP activation in infected cells is mediated by chlamydial antagonism of Hippo, we would expect that an infection-associated increase in nuclear:cytosolic YAP would be lost in cell culture conditions of minimal cell-cell contact (and, by extension, minimal YAP inhibition by Hippo). Thus, we monitored YAP responsiveness to cell seeding configurations with differing degrees of cell-cell contact. YAP nuclear incidence was not increased in mock-infected cells relative to mock-infected confluent monolayers, suggesting that residual cell-cell contact in small cell clusters maintained Hippo activity (**Supplementary Figure S4**). By contrast, nuclear:cytosolic YAP was increased in mock-infected monolayers cultured in calcium-deplete media relative those cultured in calcium-replete media (**Figures 3A, B**), confirming previous reports of adherens junction disruption being a known trigger of YAP activation, presumably through destabilization of the Hippo kinase cascade complex. Importantly, infected cells exhibited increased YAP nuclear translocation relative to mock-infected control cells in calcium-deplete conditions (**Figures 3A, B**), indicating that infection yielded an additional level of activation over calcium depletion alone.

This result suggests an activating process independent of the known YAP regulatory mechanism associated with adherens junctions, *i.e.* Hippo kinase cascade-dependent inhibition by phosphorylation at residue S127. Therefore, we sought to confirm independence of infection-associated YAP activation from the adherens junction-related regulatory mechanism. Downstream of Hippo complex formation at cell-cell contacts, complex members LATS1/2 inhibit YAP *via* phosphorylation at S127. YAP phosphorylated at this residue is bound by the adaptor protein 14-3-3, retaining YAP in the cytosol (Zhao et al., 2007; Piccolo et al., 2014). We collected whole cell lysates from mock- and *Chlamydia*-infected confluent End1 monolayers, assaying YAP S127 phosphorylation *via* Western blotting. *Chlamydia*-infected End1 monolayers exhibited equivalent levels of S127 phosphorylation relative to mock-infected controls (**Figures 3C, D**).

An alternative means of YAP activation was recently described, involving phosphorylation of YAP at Y357 (Smoot et al., 2018). While the specific mechanism of this effect remains unclear, it is suggested that YAP phosphorylation at Y357 and other tyrosine residues may promote YAP translocation into the nucleus (Elbediwy et al., 2018), or YAP retention in the nuclear compartment (Sugihara et al., 2018). To determine whether *Chlamydia* promotes YAP activation *via* this alternative mechanism, we assayed YAP phosphorylation at Y357 in infected End1 monolayers. In contrast to levels of S127-pYAP, Y357 phosphorylation was significantly increased in infected whole cell lysates relative to mock-infected controls (**Figures 3C, E**). This result was consistent with mock-infected cells treated with sodium orthovanadate (Na_3_VO_4_), a tyrosine phosphatase inhibitor that serves as a positive control for increased YAP tyrosine phosphorylation (**Figures 3C, E**) (Sugihara et al., 2018). Intriguingly, unlike nuclear incidence of YAP during infection, Y357 phosphorylation was not reduced by 1 hour of chloramphenicol treatment, suggesting that chlamydial enhancement of Y357 phosphorylation is less sensitive to inhibition of bacterial protein synthesis. Nevertheless, an 8-hour chloramphenicol treatment attenuated infection-associated Y357 phosphorylation, with attendant reduction in chlamydial Hsp60 expression indicating that this phenotype is sensitive to a delay in chlamydial development (**Figures 3F, G**). Collectively, these data demonstrate that infection-dependent YAP nuclear translocation bypasses S127 phospho-inhibition, instead correlating with increased YAP phosphorylation at Y357.

### 3.4 Chlamydial YAP activation requires the activity of host Abl kinase

**Figure 4:**
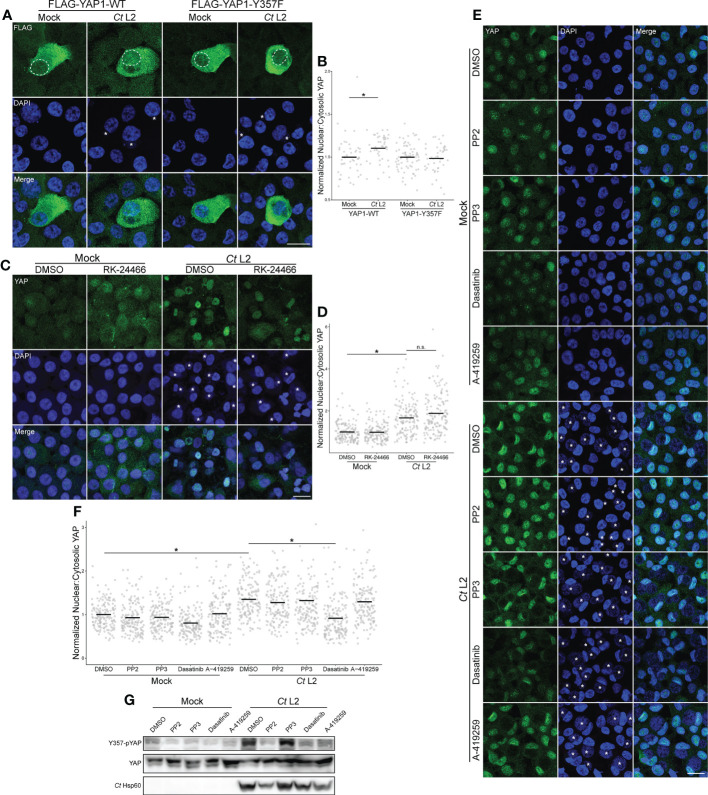
Infection-induced phosphorylation at Y357 is required for YAP nuclear translocation. **(A)** Representative micrographs of the translocation of FLAG-tagged YAP wild-type or the phospho-dead Y357F mutant (green) into the nuclei (blue) of confluent mock- and *Ct* L2-infected End1 cells at 24 hpi. Asterisks: chlamydial inclusions, scale bar: 20 μm, dotted lines: nuclear area of transfected cells. **(B)** Quantification of YAP nuclear translocation in **(A)**. n = 3 biological replicates, 50 cells measured per sample. Black bars: group means; asterisks: p-values ≤ 0.05, using pairwise Wilcoxon rank sum tests and Bonferroni’s correction for multiple comparisons. **(C)** Representative micrographs of YAP translocation at 24 hpi in confluent mock- and *Ct* L2-infected End1 cells treated with the Lck inhibitor RK-24466 (10 nM for 24 h), or DMSO. Asterisks: chlamydial inclusions, scale bar: 20 μm. **(D)** Quantification of YAP nuclear translocation in **(C)**. n = 3 biological replicates, 50 cells measured per sample. Black bars: group means; asterisks: p-values ≤ 0.05, using pairwise Wilcoxon rank sum tests and Bonferroni’s correction for multiple comparisons. **(E)** Representative micrographs of YAP translocation at 24 hpi in confluent mock- and *Ct* L2-infected End1 cells treated with the Src-family kinase (SFK) inhibitor PP2 (10 μM for 24 h), the inactive PP2 variant PP3 (10 μM for 24 h), the SFK/Abl inhibitor dasatinib (1 μM for 1 h), the SFK inhibitor A-419259 (1 μM for 5h), or DMSO. Asterisks: chlamydial inclusions, scale bar: 20 μm. **(F)** Quantification of YAP nuclear translocation in **(E)**. n = 3 biological replicates, 50 cells measured per sample. Black bars: group means; asterisks: p-values ≤ 0.05, using pairwise Wilcoxon rank sum tests and Bonferroni’s correction for multiple comparisons. **(G)** Representative Western blot of YAP tyrosine phosphorylation of mock- and *Ct* L2-infected End1 cells at 24 hpi, treated with the SFK inhibitor PP2 (10 μM for 24 h), the inactive PP2 variant PP3 (10 μM for 24 h), the SFK/Abl inhibitor dasatinib (1 μM for 1 h), the SFK inhibitor A-419259 (1 μM for 5h), or DMSO.

To determine the significance of Y357 phosphorylation, we ectopically expressed FLAG-tagged Y357F-mutant YAP in infected End1s *via* transient transfection, and compared nuclear incidence to an equivalent mock-infected control. Infected cells expressing the wild-type construct exhibited an increased ratio of nuclear:cytosolic YAP relative to mock-infected cells. However, infection did not increase nuclear incidence of the phospho-dead YAP-Y357F mutant, with infected cells exhibiting equivalent nuclear:cytosolic YAP to mock-infected cells expressing the same construct (**Figures 4A, B**). This result suggests that loss of Y357 ablates YAP nuclear translocation mediated by infection, highlighting involvement of an alternative strategy.

YAP phosphorylation at Y357 phosphorylation has been shown to be sensitive to inhibition of the Src family of tyrosine kinases; knockdown of Lck in particular is reported to decrease this phosphorylated species of YAP (Smoot et al., 2018; Sugihara et al., 2018). We therefore opted to assay YAP nuclear incidence in infected End1 monolayers treated with the Lck-specific small molecule inhibitor RK-24466. Surprisingly, the ratio of nuclear:cytoplasmic YAP in infected cells was unaffected by Lck inhibition (**Figures 4C, D**), suggesting a different mechanism of Y357 phosphorylation modulated by *Chlamydia.* We then assayed YAP nuclear incidence of infected cells treated with the pan-Src-family kinase (SFK) inhibitors PP2 and A-419259, as well as the dual-acting SFK/Abl inhibitor dasatinib (Wilson et al., 2002; Pene-Dumitrescu et al., 2008; Sugihara et al., 2018). In contrast with PP2 and A-419259 treatment, serovar L2-infected cells treated with dasatinib exhibited significant attenuation of YAP nuclear incidence relative to DMSO-treated cells, suggesting that infection-enhanced YAP nuclear incidence is mediated by Abl (**Figures 4E, F**). However, YAP phosphorylation at Y357 in infected cells was severely attenuated by treatment with all three inhibitors (**Figure 4G**), in accordance with studies directly implicating SFKs in YAP tyrosine phosphorylation (Li et al., 2016; Sugihara et al., 2018). Taken together, these data indicate that *Chlamydia*-mediated induction of YAP nuclear translocation requires host Abl activity, apparently altering translocation independent of Y357 phosphorylation.

## 4 Discussion

In this report, we present a novel mechanism for *Chlamydia*-directed modulation of host gene expression. Infection of primary and immortalized epithelial cells with *C. trachomatis* serovar L2 resulted in a host transcriptome consistent with induction of the transcriptional coactivator YAP and its DNA-binding partners. Consistent with this observation, infected cells also exhibit increased YAP nuclear incidence. However, only an approximate two-thirds of the YAP regulon were induced during infection, indicating more complex regulation of YAP or the existence of alternative transcriptional regulation *via* presence of additional transcription factor binding sites. Nevertheless, those genes activated by infection were shown to be dependent on YAP *via* siRNA knockdown and pharmacological inhibition. Enhanced YAP activation is first detectable at 18 hours post-infection; critically, this phenotype was attenuated by inhibition of bacterial protein synthesis, indicating YAP activation is a pathogen-directed effect of infection. We sought to identify a mechanism for this phenotype beginning with investigations into Hippo kinase cascade-associated phosphoinhibition of YAP as residue S127. First, the activity of the Hippo kinase cascade was reduced by focusing on cells seeded in a manner that minimized cell-cell contact. Increased nuclear YAP in infected cells was maintained in these conditions, suggesting chlamydial modulation of YAP bypasses the regulatory effect of Hippo. Accordingly, YAP phosphorylation at S127 was unchanged by infection; instead, YAP activation in infected cells was associated with phosphorylation at Y357. Y357F substitution abolished phosphorylation and led to decreased YAP nuclear incidence in infected End1 monolayers, suggesting this post-translational modification is required for chlamydial YAP nuclear translocation. However, inhibition of Y357 phosphorylation *via* treatment with the SFK inhibitors PP2 and A-419259 did not attenuate infection-associated enhancement of nuclear YAP. By contrast, simultaneous inhibition of Abl and SFKs *via* the dual-acting inhibitor dasatinib significantly reduced nuclear YAP in *Chlamydia*-infected cells, suggesting that pathogen-directed YAP nuclear translocation, but not Y357 phosphorylation requires host Abl activity. Collectively, these data point to an alternative and relatively unexplored mode of YAP activation by *C. trachomatis*.

The observation that SFK-mediated phosphorylation of YAP at Y357 is dispensable to the increased nuclear incidence of YAP observed in infected cells is seemingly in conflict with our finding that nuclear incidence of a phosphodead YAP mutant is reduced in infected cells relative to a wild-type control. This discrepancy implies that Y357 phosphorylation is necessary, but not sufficient to promote YAP nuclear localization. It is possible that alternative phosphorylation event(s) mediated by Abl kinase could be functionally redundant. Importantly, phosphorylation of YAP at Y341 and Y394 has also been reported (Li et al., 2016), with tyrosine-to-phenylalanine mutation of all three tyrosine residues impacting YAP nuclear translocation in sparse culture of Caco2 cells (Elbediwy et al., 2018). However, the specific role of phosphorylation at each site in regulating YAP nuclear translocation is unclear, as is the degree to which Y341/Y394 phosphorylation depends upon Abl activity. An alternative explanation reconciling these data is that *C. trachomatis* enhances retention of YAP in the nuclear compartment. Given that the host cell pool of Y357-pYAP presumably diminishes gradually after SFK inhibitor treatment (via the action of Y357- and/or YAP targeting phosphatases/proteases), *Chlamydia* may thus maintain YAP activation by reducing the rate of YAP export from the nucleus, even if Y357 phosphorylation is initially required for YAP entry into the nuclear compartment. Our observation that one hour of inhibition of chlamydial protein synthesis attenuates YAP nuclear translocation without an attendant effect on infection-associated Y357 phosphorylation would seem to support this hypothesis, in suggesting that *Chlamydia* enhances YAP nuclear translocation *via* multiple, complementary mechanisms with varying reliance upon Y357. Importantly, the mechanistic role of Y357 in regulating YAP is incompletely understood, with phosphorylation at this site enhancing YAP nuclear incidence, retention, or transcriptional activity depending on cell type and culture conditions (Ege et al., 2018; Elbediwy et al., 2018; Smoot et al., 2018; Sugihara et al., 2018). Ultimately, further examination of YAP nuclear import/export kinetics and the identification of alternative phosphorylation events in infected cells is required to determine how *C. trachomatis* infection modulates the function of this transcription factor.

Our bulk RNA-sequencing results indicate a substantial component of the YAP-responsive gene set identified *via* ChEA was downregulated in response to infection. While YAP is conventionally understood to enhance target gene expression when cooperating with TEAD1-4 and other transcription factors (Zagurovskaya et al., 2009; Zanconato et al., 2015; Szeto et al., 2016), its capacity to repress transcription has also been demonstrated (Kim et al., 2015). Repression of the cell cycle regulator p27 was recently shown to require binding of YAP alongside the transcriptional repressors YY1 and EZH2 (Hoxha et al., 2020); YAP-mediated recruitment of transcriptional repressors may similarly drive the reduced expression of certain YAP target genes during infection. YAP transcriptional coactivation can also indirectly facilitate repression, such as how induction of VGLL3 expression leads to VGLL3 binding to the ESR1 promoter, recruiting a NCOR2/SMRT complex to inhibit ESR1 transcription (Ma et al., 2022). The expression of individual genes in the putative YAP regulon of infected cells is likely driven by a complex interplay of multiple transcription factors. Indeed, this dynamic may explain why infection induced one canonical YAP target (CTGF) without also inducing another (CYR61); CYR61 is inhibited by the transcriptional repressor FOXO3 and the histone deacetylase HDAC5 (Lee et al., 2007; Tsou et al., 2016), both of which are induced in our bulk RNA-sequencing of *Chlamydia*-infected End1 cells (FDRP ≤ 0.05, FC: 1.23 and 1.58, respectively). Ultimately, future work to define the true extent of the *Chlamydia*-induced YAP regulon will require comparing the infected host transcriptome to an equivalent infection in a YAP-negative background. Given that the incomplete siRNA-mediated knockdown we report here is consistent with past use of siRNA in End1s (Govender et al., 2014), generating a CRISPR/Cas9-mediated YAP-knockout from an alternative cell line may be necessary.

Our observation of *Chlamydia*-directed induction of YAP activity is consistent with prior reports of YAP modulation by other bacterial pathogens. The Gram-negative pathogen *Helicobacter pylori* was recently shown to enhance YAP expression through the action of the effector CagA (Li et al., 2018). It is unclear how CagA interacts with the steady-state inhibition of YAP by the Hippo kinase cascade; given the prior finding that CagA destabilizes host cell-cell junctions – a common site of Hippo kinase complex recruitment – one possible explanation is that CagA antagonizes Hippo-mediated YAP inhibition by indirectly preventing stabilization of the Hippo complex (Song et al., 2013). Alternatively, the observation that YAP expression is increased by CagA in a dose-dependent fashion suggests that *H. pylori* infection may instead circumvent Hippo inhibition by overwhelming either Hippo’s capacity to phospho-inhibit YAP or 14-3-3’s capacity to sequester serine-phosphorylated YAP in the cytoplasm (Li et al., 2018). The observation that *H. pylori* enhances EMT in gastric epithelia in a CagA-dependent fashion suggests a potential for pathogen-directed YAP activation to have downstream effects on pathology, such as gastric cancer predisposition (Song et al., 2013; Li et al., 2018).

Our finding that chlamydial YAP activation is sensitive to inhibition of bacterial protein synthesis indicates that one or more chlamydial effectors facilitate YAP activation. One possible candidate for this effect is the chlamydial inclusion protein IncG, which has been previously shown to interact with host 14-3-3β (Scidmore and Hackstadt, 2001). 14-3-3 family proteins are known to act downstream of Hippo-mediated YAP inhibition by binding and sequestering serine-phosphorylated YAP in the cytosol (Basu et al., 2003; Zhao et al., 2007). While the specific role of 14-3-3β and other isoforms in this effect is unclear, sequestration of 14-3-3β at the inclusion surface by IncG has the potential to decouple YAP from Hippo-mediated phospho-inhibition at S127. Indeed, a recent report indicated 14-3-3β/ϵ localize to the inclusion surface *via* binding to the inclusion membrane protein InaC (Kokes et al., 2015), suggesting that 14-3-3 sequestration (and, by extension, downstream effects on YAP activation) may be targeted by *C. trachomatis* in multiple ways. Given that chlamydial YAP activation is demonstrably sensitive to inhibition of Abl, screening for chlamydial modulators of Abl or signaling pathways that lead to increased Abl activity may identify additional YAP-activating effectors. Importantly, Abl was identified in an RNA interference screen for host factors involved in invasion by the related species *C. muridarum* (Elwell et al., 2008), and has been demonstrated to phosphorylate the chlamydial invasion effector TarP in both species (Elwell et al., 2008; Mehlitz et al., 2008). While it is unclear the degree to which invasion-associated Abl activity influences the YAP activation during midcycle infection we report here, these data collectively illustrate a need to identify Abl-modulating chlamydial effectors.

Although we do not observe chlamydial inhibition of the Hippo kinase cascade at the level of YAP S127 phosphorylation, we cannot entirely dismiss a role for this negative regulator in determining levels of nuclear YAP during infection. The recent report of chlamydial disruption of cell-cell junctions in an organoid model of infection (Dolat and Valdivia, 2021) suggests that YAP inhibition by Hippo may be relevant in three-dimensional models of infection. Our laboratory has previously reported on chlamydial modulation of focal adhesion kinase (FAK) (Thwaites et al., 2014; Pedrosa et al., 2020); given that FAK has been shown to antagonize Hippo-mediated YAP phosphorylation at S397 (Hu et al., 2017), infection may potentially impact YAP inhibition by Hippo in this way as well. Though beyond the scope of this initial study, further investigation of chlamydial YAP activation will likely require use of three-dimensional culture models to determine which of the extensive portfolio of subcellular structures and mechanical inputs co-opted by the pathogen ultimately drive enhancement of YAP activity during infection.

Our results illustrate a mechanism by which chlamydial infection promotes YAP-dependent gene expression in the host; however, the benefit of this interaction to the pathogen is unclear. YAP nuclear translocation and subsequent increase in target gene expression in infected epithelial cells can be viewed as a downstream process relevant to pathogenesis, but not necessarily one beneficial to chlamydial growth in host cells. Given past work demonstrating *Chlamydia*-infected cells exhibit increased expression of genes encoding pro-fibrotic cytokines and ECM components (Humphrys et al., 2013; Porcella et al., 2015), and our own observation that infection induces expression of fibroblast-activating signaling factors (CTGF, INHBA, BMP2), the potential effect of infection-mediated YAP activation on *Chlamydia*-associated scarring warrants further study. Fibroblasts in particular are understood to play a critical part in other types of scar-forming disease (Kendall and Feghali-Bostwick, 2014); however, their contribution to chlamydial fibrosis has yet to be fully assessed. Understanding the yet uncharacterized effect of *Chlamydia*-infected cells on fibroblasts and other uninfected cell types will likely be critical to elucidating mechanisms of chlamydial fibrosis *in vivo*, as well as contextualizing the broader role of YAP induction in chlamydial pathogenesis.

## Data availability statement

The bulk RNA-sequencing data included in this paper is now publicly available via the Gene Expression Omnibus (GEO), accession number GSE180784. A link to the dataset is provided below: https://www.ncbi.nlm.nih.gov/geo/query/acc.cgi?acc=GSE180784.

## Author contributions

Conceptualization, LC and RC; Methodology, LC, AB, and RC; Validation, LC and AB; Formal Analysis, LC; Investigation, LC and AB; Resources, RC; Data Curation, LC; Writing – Original Draft, LC; Writing – Review & Editing, RC and AB; Visualization, LC; Supervision, RC; Project Administration, RC; Funding Acquisition, RC. All authors contributed to the article and approved the submitted version.
