# Supplementary material for: Chlamydia trachomatis induces the transcriptional activity of host YAP in a Hippo-independent fashion
Source: Front Cell Infect Microbiol. 2023 Feb 27;13:1098420. doi: 10.3389/fcimb.2023.1098420 (PMC10008951; doi:10.3389/fcimb.2023.1098420)
Supplement: Supplementary file 5 [file DataSheet_1.pdf]

Supplementary Material

1 Supplementary Data

Supplementary Data S1. Summary of differentially expressed genes detected in bulk RNA-sequencing of mock- and Ct serovar L2-infected End1/E6E7 immortalized epithelial cells (End1s). All fold-changes expressed relative to mock-infected control. See enclosed file: "Supplementary Data S1.xlsx".

Supplementary Data S2. Summary of the cross-referencing of Table S1 data with the ChIP Enrichment Analysis (ChEA) database of transcription factor target genes. Individual target list mappings for each transcription factor are available on request. See enclosed file: "Supplementary Data S2.xlsx".

Supplementary Data S3. Summary of differentially expressed genes detected in bulk RNA-sequencing of mock- and Ct serovar L2-infected primary human cervical epithelial cells (HCECs). All fold-changes expressed relative to mock-infected control. See enclosed file: "Supplementary Data S3.xlsx".

Supplementary Data S4. Summary of differentially expressed YAP target genes (as identified by ChEA) detected in bulk RNA-sequencing of mock- and Ct serovar L2-infected End1s and HCECs. All fold-changes expressed relative to each infection's respective mock-infected control. See enclosed file: "Supplementary Data S4.xlsx".

1.1 Supplementary Figures

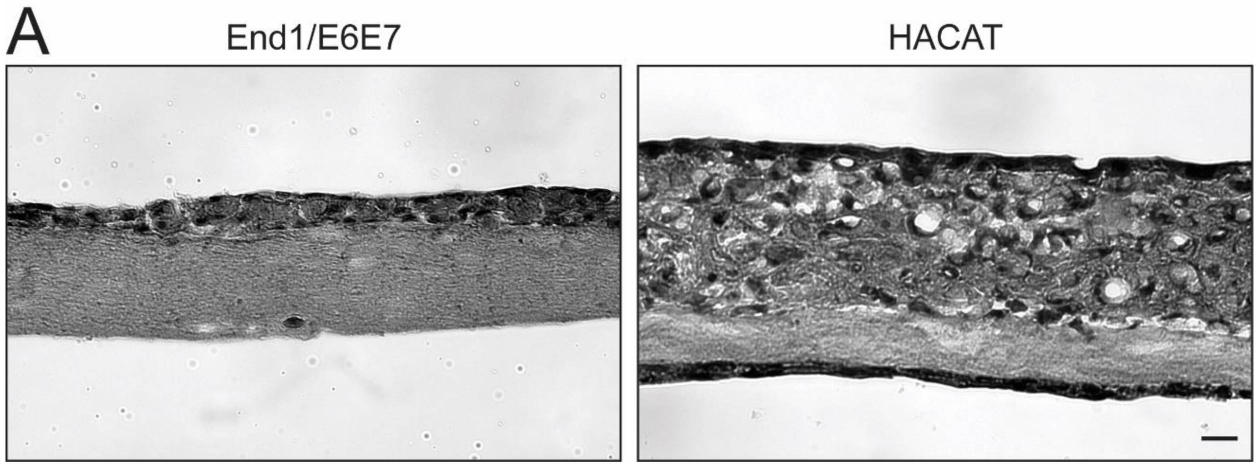

Supplementary Figure S1. End1 E6/E7-immortalized epithelial cells in organotypic culture do not exhibit morphology consistent with malignant HPV-associated transformation.
(A) Greyscale, bright-field micrograph of hematoxylin/eosin-stained 20 μm sections of End1 (left) and HaCaT (right) RAFT cultures. Scale bar: 20 μm.

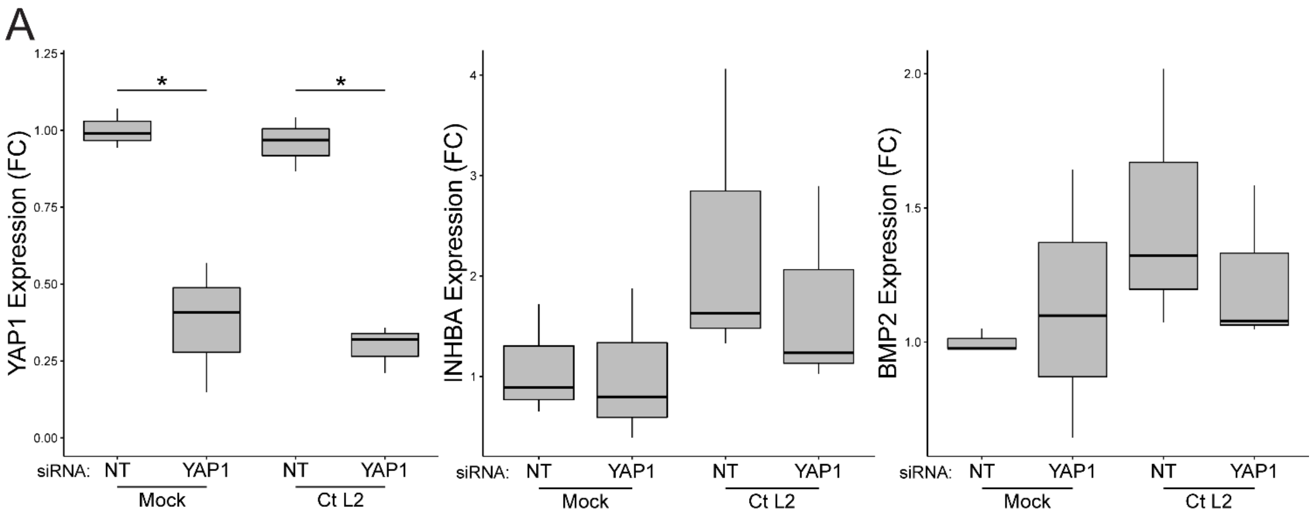

Supplementary Figure S2. siRNA-mediated YAP knockdown in *Chlamydia*-infected End1 cells. (A) Expression of YAP, INHBA, and BMP2 at 24 hpi in mock- and *Ct* L2-infected End1 cells transfected with non-targeting (NT) or YAP1-targeting siRNA (10 nM for 24 h prior to infection), as measured by RT-qPCR. n = 3 biological replicates; fold changes are relative to mean expression of the mock-infected and untreated control. Whiskers: minimum to maximum; asterisks: p-values ≤ 0.05, using pairwise Student's t-tests and Bonferroni's correction for multiple comparisons.

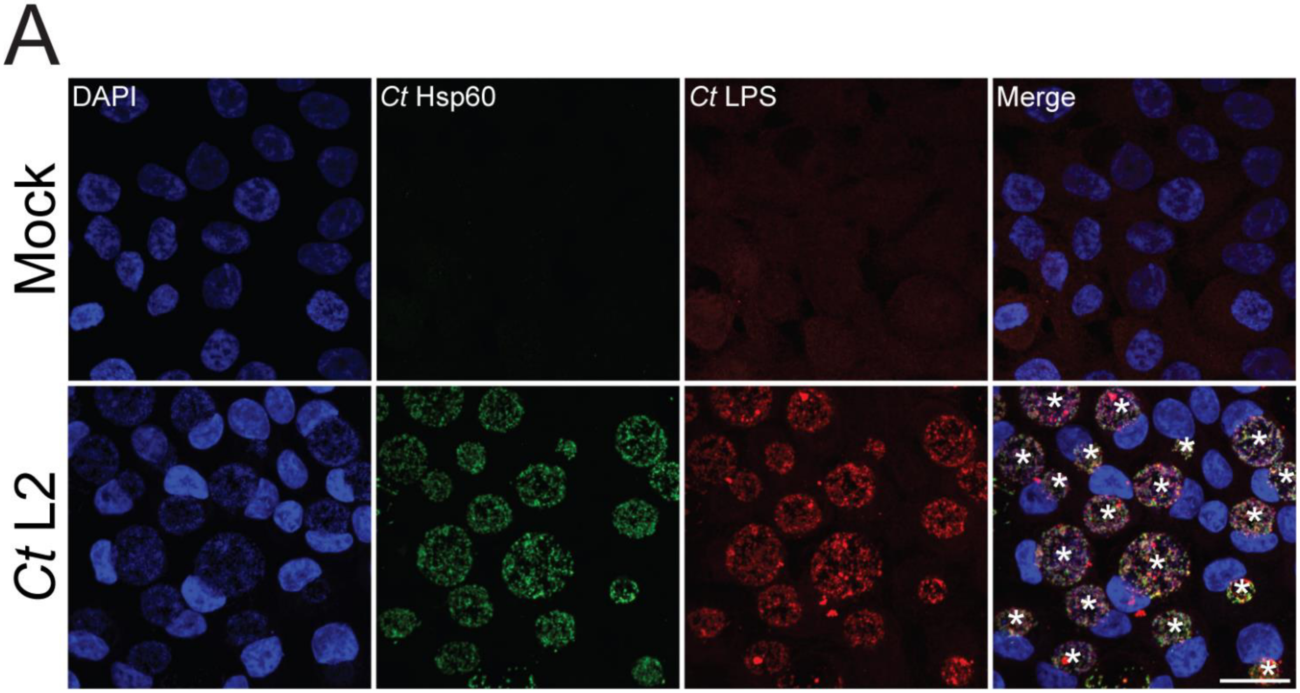

Supplementary Figure S3. *Chlamydia* infection presents with a DAPI-positive inclusion.
(A) Representative micrographs of chlamydial inclusions stained with DAPI (blue), *C. trachomatis* Hsp60 (green), and *C. trachomatis* LPS (red) at 24 hpi in confluent mock- and *Ct* L2-infected End1 cells. Asterisks: chlamydial inclusions, scale bar: 20 µm.

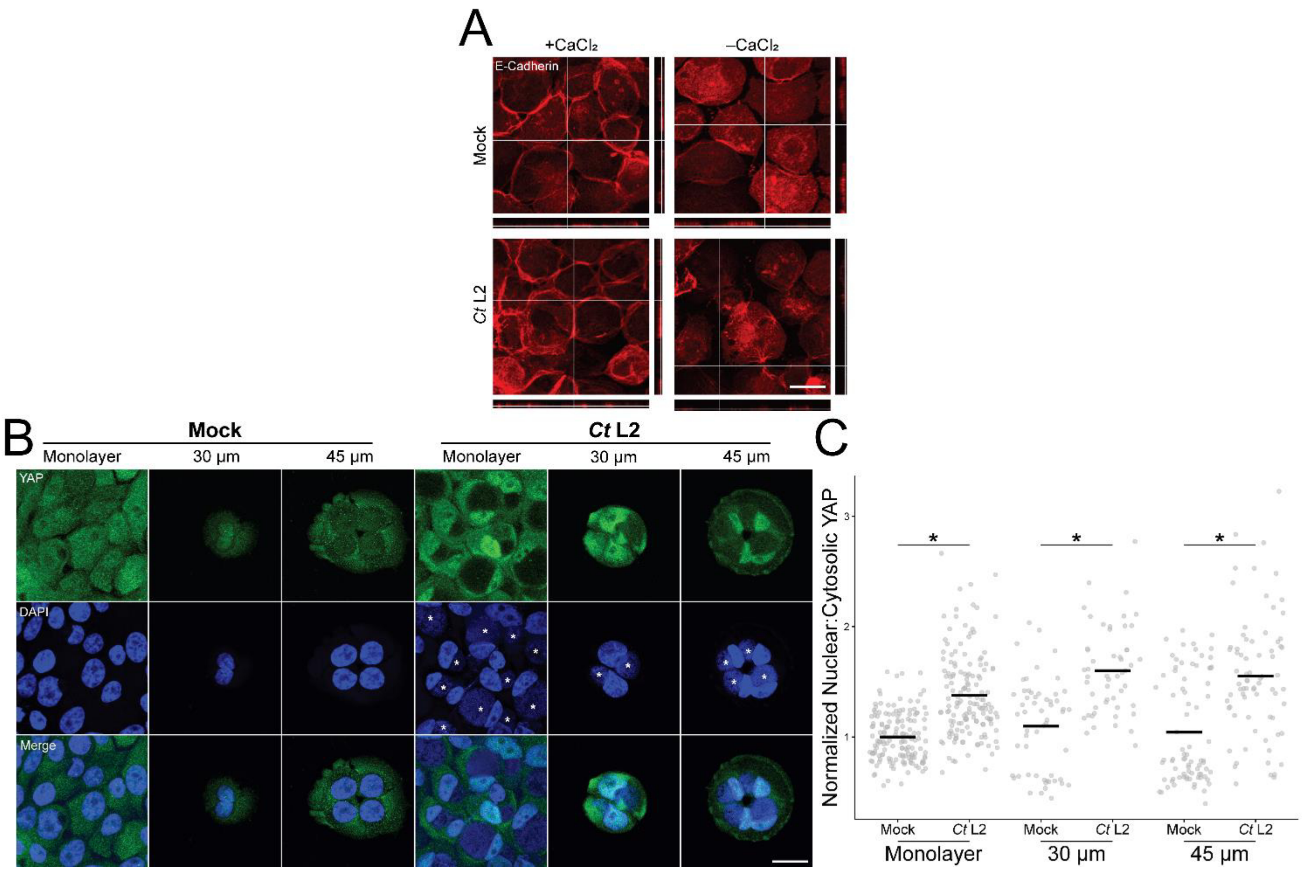

Supplementary Figure S4. *Chlamydia* infection enhances YAP nuclear translocation in conditions of Hippo attenuation.

(A) Representative micrographs of E-cadherin (red) localization at 24 hpi in confluent mock- and *Ct* L2-infected End1 cells cultured in calcium-replete (+$CaCl_2$, 0.4 mM) or calcium-deplete (-$CaCl_2$) keratinocyte serum-free media. Right sidebar: ZY-plane orthogonal view; bottom sidebar: XZ-plane orthogonal view; scale bar: 20 µm.

(B) Representative micrographs of YAP (green) translocation into the nuclei (blue) at 24 hpi of mock- and *Ct* L2-infected End1 cells cultured in confluent monolayers, or in small cell clusters using 30 µm (1-2 cells) and 45 µm (2-4 cells) micropatterns. Asterisks: chlamydial inclusions; scale bar: 20 µm.

(C) Quantification of YAP nuclear translocation in (B). n = 3 biological replicates, 50 cells measured per sample. Black bars: group means; asterisks: p-values ≤ 0.05, using pairwise Wilcoxon rank sum tests and Bonferroni's correction for multiple comparisons.
